# The Effect of Personality, Disability, and Family Functioning on Burnout among Mothers of Children with Autism: A Path Analysis

**DOI:** 10.3390/ijerph19031187

**Published:** 2022-01-21

**Authors:** Małgorzata Sekułowicz, Piotr Kwiatkowski, Iris Manor-Binyamini, Krystyna Boroń-Krupińska, Błażej Cieślik

**Affiliations:** 1 Faculty of Physical Education and Sport, Wroclaw University of Health and Sport Sciences, 51-612 Wroclaw, Poland; malgorzata.sekulowicz@awf.wroc.pl (M.S.); krystyna.boron@awf.wroc.pl (K.B.-K.); 2 Pedagogy Institute, University of Wrocław, 50-137 Wroclaw, Poland; piotr.kwiatkowski@uwr.edu.pl; 3 Faculty of Medicine, Technion, Haifa 31096, Israel; iris.manorbinyamini@gmail.com; 4 Faculty of Health Sciences, Jan Długosz University in Częstochowa, 42-200 Czestochowa, Poland

**Keywords:** maternal burnout, family, personality traits, autism

## Abstract

This path analysis of mothers of children with autism aimed to investigate the relationship between maternal burnout and the mother’s subjective reporting of difficulty in childcare, family function, and personality traits. A total of 410 mothers of children with autism (mean age 39.03, *SD* 7.42) completed four questionnaires: Parental Burnout Measure (PBM-12), International Personality Item Pool—Big Five Markers (IPIP-BFM-20), Flexibility and Cohesion Evaluation Scales (FACES-IV), and a survey on childcare difficulties. Path analysis using two predetermined models was used to examine the interrelations. Both models fit the empirical data equally with a Root Mean Square Error of Approximation (RMSEA) index of 0.000 and a 90% confidence interval (model 1: 0.000–0.052; model 2: 0.000–0.059). Path analysis revealed similar fit indexes for both models: (a) burnout is a mediator between exogenous variables and family functioning, and (b) family functioning is an indirect mediator between exogenous variables and burnout. These findings suggest that increased maternal emotional instability (neuroticism) and conscientiousness can lead to increased family communication problems, which may further lead to a breakdown of the equilibrium in the family system, resulting in the mother’s dissatisfaction with family life and a consequent increased risk of maternal burnout.

## 1. Introduction

Parenting is a treasured experience but also a very demanding one. In extreme cases, physical and emotional exhaustion can lead to parental burnout. This refers to a specific syndrome of exhaustion related to prolonged situations of emotional imbalance where the burden of perceived stress exceeds one’s personal coping resources [1,2]. It encompasses three dimensions: emotional exhaustion (related to one’s parental role), emotional distancing (the tendency to distance oneself from one’s children), and the feeling of a lack of personal accomplishment (reflecting a sense of ineffectiveness in one’s parental role; [3,4]. Research on parental burnout is still in its infancy, but studies to date have shown that it can be reliably measured in both mothers and fathers and that it has been shown to occur in between 8% and 36% of parents, depending on the characteristic of parents studied [3,5]. According to the most conservative point prevalence estimates, at least 3.5 million American parents currently suffer from parental burnout [6]. A study in Europe revealed that 2–12% of parents might have experienced parental burnout [3].

Because burnout is the result of an ongoing, significant imbalance of demands for resources, Schaufeli et al. (2009) and Lindström et al. (2011) suggested that relevant risk factors for burnout theoretically involve factors in the microsystem, mesosystem, and macrosystem and that the existence of stable traits would influence one’s vulnerability to parental burnout [5,7]. Parents are at greatest risk when they aim to be “perfect” parents [8]; suffer from neurosis, lack emotion, and/or lack stress-management abilities [9]; lack emotional or practical support from the co-parent or the social network [4,10]; have poor child-rearing practices [4]; have children with special needs that interfere with family life [11]; and/or work part-time [5]. Particular attention should be paid to neuroticism, which is one of the most important risk factors in parental burnout [2]. According to researchers, emotional instability (i.e., neuroticism) encompasses emotional control and impulse control behavior, and emotionally unstable parents respond more intensely to life events [12].

Two other important factors that may lead to parental burnout are conscientiousness and the specific characteristics of the child. Conscientiousness refers to self-discipline, order, and planning—ostensibly positive features—but also includes a propensity for meticulousness and obsessiveness. Individuals with higher levels of conscientiousness report fewer negative effects, especially guilt, and are better able automatically to down-regulate negative effects [12]. The child’s particular characteristics determine the intensity of the demands on parents and may increase the risk for burnout, especially in a child with behavioral, emotional, or learning disorders [13] or with a physical disability or chronic illness [14,15]. Parents caring for children with disabilities appear to be at a greater risk of burnout [3,11].

Because the family is where parenting takes place, three factors relating to family function may play a role in burnout by increasing/decreasing demands on parents or the resources they require for parenting. These are marital satisfaction, good co-parenting, and organization in the family. As already suggested, a nurturing relationship, communication, and happiness with one’s partner (i.e., greater marital satisfaction) reduce parental burnout [5]. A co-parent who agrees with one’s educational goals and practices cooperates in making parenting decisions and values the other as a parent should also reduce burnout, especially as it has recently been shown to be related to reduced parenting stress [16]. By contrast, disorganization in the family (i.e., mess, agitation, noise, and the absence of routines) may be related to increased burnout [17].

When raising children with disabilities, internal and external patterns of communication in the family system may be enhanced or adapted, and roles often need to be reorganized to meet the demands of caregiving [18]. Disorder or lack of consistency and flexibility in the family can lead to parental burnout. The family function is well described by Olson (2011), who described his revised circumplex model of marital and family systems [19]. The main assumption here is that balanced levels of cohesion and flexibility (low to high levels) are most conducive to a healthy family function, while unbalanced levels of cohesion and flexibility (very low or very high levels) are associated with problematic family function. Moreover, unbalanced cohesion can consist of poor involvement on the part of the family (*Disengaged*) or too much involvement (*Enmeshed*). Unbalanced flexibility can consist of excessive emphasis placed on compliance with rules and role stability (*Rigid*) or a lack of clear rules and division of roles (*Chaotic*) [19]. There are five levels of flexibility: the lowest level (rigidity), three central levels referred to as balanced and reflecting healthier functional levels, and the highest level (chaos). The extreme levels: rigidity (very low) and chaos (very high) are recognized as imbalanced [20]. Research by Mikolajczak and Roskam (2018) conducted into families operating under the threat of parental burnout suggests that it results from a chronic imbalance between demands (risk factors) and resources (protection factors). The stress-increasing factors include “parental perfectionism, low emotional intelligence, poor child-rearing practices, countless parental duties and chores, lack of support from the other parent, lack of external support (family support, nurseries, etc.)” [2]. The protection factors that significantly decrease parental stress include ”parental self-compassion, high emotional intelligence, good child-rearing practices, time for leisure, positive co-parenting, external support, etc.” [2]. The results obtained by Mikolajczak and Roskam (2018) may indicate disturbed family cohesion and flexibility, which may lead to parental burnout [2].

Until recently, research on parental exhaustion was exclusively concerned with parents of chronically ill children [15,21], and there is scant research on maternal burnout in the context of rearing and caring for children with disabilities, particularly those with autism. Autism is a pervasive developmental condition characterized by a triad of issues, namely, deficiencies in social reciprocity and communication, repetitive behaviors, and obsessive interests. These present the parent of a child with autism with a unique set of challenges that can adversely affect the parents’ wellbeing, and the ongoing stress associated with raising such a child may lead to parental burnout [17].

While the growing literature on the subject investigates parental burnout, the experience of parents caring for children with chronic illnesses or disabilities is a special case. Parents caring for children with physical or mental issues appear to be at a greater risk of burnout [3,11]. In a study by Hubert and Aujoulat (2018), burnout families with sick children “reported developing attitudes of self-blame while pointing to their own personality and identity as a mother to explain the painful situation in which they described themselves both as victims and perpetrators. Interestingly, none of the women subjected to the study reported regretting motherhood” [22]. Parents of children with autism are a group particularly vulnerable to burnout due to their children’s developmental difficulties. According to Pisula and Porebowicz-Dorsmann (2017), “*one of the key variables of parental adjustment is family functioning, counted among the resources facilitating adjustment*”. Factors such as “*family commitment, challenge, cohesion, expression, and marital support, are recognized as the predictors of family quality of life*” [23]. The authors point out that most studies on the relation between family functioning and the level of adjustment of parents raising children with ASD are conducted on mothers. This is the reason why considering the parents’ gender and their involvement in caretaking seems imperative [23].

Although these seminal studies shed light on the importance of focusing on parental burnout, the topic requires further investigation. Many questions need to be addressed, but one of the most pressing is identifying burnout antecedents/risk factors, for this is a prerequisite to developing suitable action in terms of both prevention and intervention. Therefore, the present study aimed to use path analysis to investigate the relationship between parental burnout in mothers of children with autism and their subjective view of their difficulty in childcare, family functioning, and personality traits. In order to achieve this, we compared the suitability of two path interrelation models based on the variables mentioned above. The models differ with respect to the direction of the hypothetical cause-and-effect relationship between parental burnout and the dimensions of family function. We tested these two contrasting models because we theorized that causal relationships between family function and burnout could work in either direction. As a result, in model 1, burnout has the status of an exogenous variable, while in model 2, burnout is an outcome variable explained by the model.

## 2. Materials and Methods

### 2.1. Participant Recruitment and Sample Size

The participants were not randomly selected. They were recruited from the Polish population through the websites of the Organization for Parents of Children with Autism Spectrum Disorder (ASD) and online forums for parents of children with autism and asked to volunteer for an online survey. Some of the participants who were recruited were parents undergoing counseling or were in parent support groups, and some had received a link to the survey from others (snowball sampling). In total, 439 parents of children with autism responded to the survey and completed the questionnaire. Since there was an over-representation (93.5%) of mothers in the sample, the 29 fathers (6.6%) were excluded from the analysis post factum, leaving the number of participants at *n* = 410. Table 1 presents the socio-demographic characteristics of the participants.

The research complied with all the relevant national regulations, institutional policies, and tenets of the Helsinki Declaration. All study procedures were approved by the Institutional Review Board at the Wroclaw University of Health and Sport Sciences (Poland). No compensation was offered to participants. All responses were anonymous. Informed consent was obtained from all individuals included in the study.

### 2.2. Outcome Measures

#### 2.2.1. Psychological Difficulties with Childcare

This variable includes two subjectively assessed questions on the 3-point Likert scale: degree of dysfunction, that is, the mothers’ subjective assessment of their independence (impossible, seriously limited, or slightly limited), and expectation of rehabilitation (the child’s condition will deteriorate, remain unchanged, or improve). The correlation between the items was shown to be statistically significant (Spearman’s Rho = 0.21, *p* < 0.05). The internal reliability was not calculated because it is futile when calculated for two items. 

#### 2.2.2. Personality Assessment

Personality traits were assessed using the 20-item International Personality Item Pool—Big Five Markers (IPIP-BFM-20) that had been translated into Polish [24]. This questionnaire is a condensed version of the 50-item International Personality Item Pool [25]. The item selection procedure was similar to that used by Donnellan et al. (2006) in the Mini International Personality Item Pool (Mini-IPIP) [26]. The questionnaire measures five traits: extraversion, agreeableness, conscientiousness, emotional stability, and intellect/imagination. The Cronbach’s alpha coefficients for this questionnaire ranged from 0.65 to 0.78.

#### 2.2.3. Parental Burnout

Parental burnout was measured using the Polish adaptation of the Parental Burnout Measure (PBM-12) [27]. The scale contains 12 four-point Likert items and two oblique factors: exhaustion (6 items) and helplessness (6 items). Because both subscales were relatively strongly correlated (0.78), the existence of a secondary factor was suspected, and therefore the overall result of the scale, that is, the sum of both subscales, was used. As a result, a fully satisfactory Cronbach’s alpha of all burnout measures—exhaustion subscale (0.88), helplessness subscale (0.80), and PBM-12 total score (0.90)—was obtained.

#### 2.2.4. Family Equilibrium, Family Communication, and Satisfaction with Family Life

The family function was evaluated using the Polish version of the Flexibility and Cohesion Evaluation Scales (FACES-IV) [19,20]. FACES-IV comprises 62 items (with a 5-point rating scale) with eight subscales: balanced cohesion, balanced flexibility, disengagement, enmeshment, rigidity, chaos, family communication, and satisfaction with family life. These subscales form the circumplex model. The circumplex total ratios were calculated using the following formula:

$$\frac{Balanced\;Cohesion+Balanced\;Flexibility}{2}/\frac{Disengagement+Enmeshment+Rigidity+Chaos}{4}\;=\;\;2\;\times \;\frac{Balanced\;Cohesion+Balanced\;Flexibility}{Disengagement+Enmeshment+Rigidity+Chaos}$$

In the sample used, two subscales—enmeshment and rigidity—revealed an internal consistency below alpha = 0.70 (*α* = 0.63 and 0.55, respectively). Nevertheless, measuring the functionality of a family system using the circumplex total ratio may be considered to be reliable because the alpha values of the others were sufficiently high (>0.70), and the formula shows that the contribution of the two problematic components to the final result was smaller than the contribution of balanced cohesion and flexibility.

The other two FACES-IV dimensions—family communication and satisfaction with family life—comprise ten items each. Family communication is defined as positive communication skills utilized by the couple or in the family system. This dimension is viewed as a facilitating one that helps families alter their levels of cohesion and flexibility. Satisfaction with family life measures the mothers’ satisfaction with several aspects of family life. Both subscales have high internal consistency (Cronbach’s alpha > 0.90) [19,20].

### 2.3. Model Assumptions

**Figure 1:**
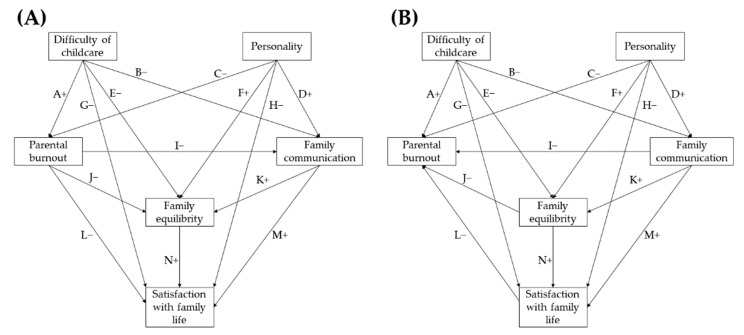
The main (**A**) and alternative (**B**) set of hypotheses models of causal correlation between difficulty of childcare, maternal personality, maternal burnout, and three features of family functionality. Sign (+ or −) are expected correlations (positive or negative).

Initially, we made three general assumptions. First, according to Olson’s (2011) model, family communication influences the balance of the family system and affects satisfaction with family life. Second, parental burnout is an exogenous variable of the psychological difficulty of caring for children with autism, the “big five” personality traits, and the functioning of the family system. Third, the difficulty of childcare and personality traits also directly affect all three dimensions of the functioning of the family system. This major model of hypothetical links between the variables is presented in the diagram below (Figure 1). The same figure also contains an alternative model. Here, the direction of the three causal relationships was modified, leading from family functionality to parental burnout.

In Figure 1, we presented all the hypotheses for the relations between the selected variables. The specific relations are marked with letters, symbols (plus or minus), and arrows. The character defines the expected correlation (positive or negative), while the arrows define causality in single relation. In the terminology of path analysis, the above Figures can be referred to as saturated models because all the variables are associated with each other (for simplicity, the relation between the difficulty of childcare and the mother’s personality was omitted, but the covariance of these variables was tested). In the figure, we presented all the hypotheses for the relations between the selected variables. The particular relations are marked with letters, symbols (plus or minus), and arrows. The character defines the expected correlation (positive or negative), while the arrows define causality in single relation. In the terminology of path analysis, the above figures are referred to as “saturated” models because all the variables are associated with each other (for simplicity, the relationship between the difficulty of childcare and the mother’s personality was omitted—the covariance of these variables will be tested). The differences between these two models refer to the relations marked with the letters I, J and L and pertain to the causal direction (the correlation marks remain unchanged). The “Personality” category includes all the features of the Big Five model as separate variables for analysis.

We tested alternative models because we assumed that the causal relationship between family function and burnout could work in either direction. If there is a link between burnout and the functioning of a working person’s family [28,29,30], it is difficult to expect burnout in an important family role as unrelated to the functioning of the family system [31]. A circular relationship is very likely. However, we could not verify this thesis because our study was not longitudinal. We could only examine both causality variants in separate analyses (each of the alternative models separately).

### 2.4. Data Analysis

Data were analyzed using SPSS 25.0 software (IBM Corp, Washington, WA, USA). The continuous variables are presented as means and standard deviations (*SD*s), and the categorical responses are presented as frequencies and percentages. Correlations were tested using Pearson’s linear correlation. Path analysis was used to evaluate the proposed relationships between parental burnout, the difficulty of childcare, personality traits, family equilibrium, family communication, and satisfaction with family life. Figure 1 shows hypothetical links between the variables for both models. Since skewing and kurtosis were more than double the standard error, we first carried out a stepwise regression analysis to eliminate the variables with low predictive power (Supplementary Material Tables S1–S4). All variables were standardized (*Z-score*), and the fit of the model was assessed using the following indexes: root mean square error of approximation (RMSEA), goodness-of-fit index (GFI), adjusted goodness-of-fit index (AGFI), comparative fit index (CFI), and critical ratio (CR).

## 3. Results

### 3.1. Participants and Characteristics

The majority of respondents possessed higher education (59%), lived in urban centers (79%), were parents raising the child (ren) together with the other biological parent (77%), and were not employed (68%). The ages of the respondents were between 18 and 67 (mean 39.03, *SD* 7.42). The ages of the children with autism were between 1 and 40 (mean 9.74, *SD* 7.41), and they were predominantly male (73%). The majority (73%) of parents chose the expectation of rehabilitation for the dynamics of the child’s disability. Significantly reduced self-care was the most frequently chosen category (49%) to describe the degree of disability of a child. Among the respondents, 9% had the status of a person with disabilities. See Table 1 for a complete breakdown.

### 3.2. Correlations in the Data Set

The analyses found that burnout was negatively correlated with extroversion (*r* = −0.32) and emotional stability (*r* = −0.68) and positively correlated with agreeableness (*r* = 0.20) and conscientiousness (*r* = 0.26). Family equilibrium, sound family communication and maternal satisfaction with family life were negatively correlated with burnout (*r* = −0.43, −0.36, −0.46, respectively) and positively correlated with extraversion (*r* = 0.20, 0.21 and 0.29, respectively) and emotional stability (*r* = 0.42, 0.31, 0.40). They were also negatively correlated with agreeableness (*r* = −0.27, −0.17, −0.22) and conscientiousness (*r* = −0.25, −0.24, −0.29). Childcare difficulties were positively correlated with burnout (*r* = 0.24) and negatively correlated with family equilibrium (*r* = −0.10) and family communication (*r* = −0.10). For all the analyses mentioned herein, *p* < 0.05. See Table 2 for the complete matrix.

### 3.3. Path Analyses

**Figure 2:**
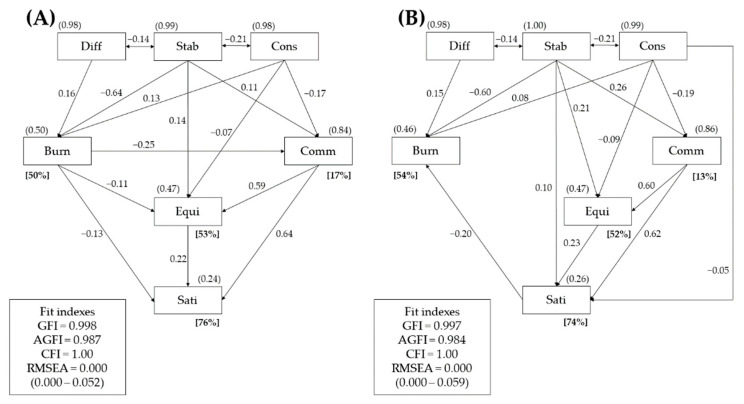
Final reduced versions of the mail (**A**) and alternative (**B**) path models for mothers of children with disabilities (*n* = 410). Abbreviations: Diff—difficult childcare; Cons—conscientiousness; Stab—emotional stability; Burn—parental burnout; Equi—family equilibrium; Comm—effective family communication; Sati—satisfaction with family.

Figure 2 illustrates final reduced versions of the two path models. The two pathway models differed, as noted above, in terms of their dependent variables: for model 1, it was satisfaction with family life, and for model 2, it was parental burnout. For each, we considered the following two sets of independent variables: (a) psychological difficulties in childcare, emotional stability, and conscientiousness (exogenous variables); and (b) maternal burnout, family communication, family balance (equilibrium), and maternal satisfaction with family life (endogenous variables). Both models proved to be equally well-fitted to the empirical data with RMSEA indexes of 0.000 with a 90% confidence interval (0.000−0.052 for reduced model A and 0.000−0.059 for reduced model B).

Model 1 indicated 11 immediate, unidirectional relationships between the variables. Positive correlations were found between the following (note that for all *β*-values given below, *p* = 0.000 except otherwise indicated): difficult childcare and maternal burnout (*β* = 0.1580), conscientiousness and maternal burnout (*β* = 0.126), emotional stability and effective family communication (*β* = 0.111, *p* = 0.075), emotional stability and family equilibrium (*β* = 0.138), effective family communication and family equilibrium (*β* = 0.590), family equilibrium and satisfaction with family life (*β* = 0.220), and effective family communication and satisfaction with family life (*β* = 0.640). Negative correlations were found between emotional stability and maternal burnout (*β* = −0.635), conscientiousness and effective family communication (*β* = −0.168, *p* = 0.001), conscientiousness and family equilibrium (*β* = −0.067, *p* = 0.040), maternal burnout and effective family communication (*β* = −0.246), maternal burnout and family equilibrium (*β* = −0.112, *p* = 0.012), and maternal burnout and satisfaction with family life (*β* = −0.128)(Figure 2).

Model 2 indicated 13 immediate, unidirectional significant correlations between the variables (*p* < −0.10; *p* = 0.000 unless noted). Positive correlations were found between difficult childcare and maternal burnout (*β* = 0.153), conscientiousness and maternal burnout (*β* = 0.082, *p* = 0.019), emotional stability and effective family communication (*β* = 0.263), emotional stability and family equilibrium (*β* = 0.211), emotional stability and satisfaction with family life (*β* = 0.100), effective family communication and family equilibrium (*β* = 0.598), family equilibrium and satisfaction with family life (*β* = 0.228), and effective family communication and satisfaction with family life (*β* = 0.624). Negative correlations were found between emotional stability and maternal burnout (*β* = −0.569), conscientiousness and effective family communication (*β* = −0.194), conscientiousness and family equilibrium (*β* = −0.087, *p* = 0.007), conscientiousness and satisfaction with family life (*β* = −0.052, *p* = 0.075), and satisfaction with family life and maternal burnout (*β* = −0.201)(Figure 2).

An analysis of the squares of multiple correlations for the four endogenous variables provided important information on the accuracy of the verified path models. While both models successfully explained maternal satisfaction with family life (model 1 showed it to be 76% and for model 2, 70% of the variance, respectively), they were less successful at explaining burnout (50% and 54% of the variance) and a balanced family system (53% and 52% of the variance). The least explained variable in the models was the quality of family communication (17% and 13% of the variance), which was shown to be correlated with personality traits—emotional stability (a positive correlation) and conscientiousness (a negative correlation). Model 1 showed that effective communication was inversely related to the degree of maternal burnout. This explains the slightly higher ratio in the explained variation in the variable in that model (Figure 2).

## 4. Discussion

### 4.1. Childcare Difficulty and Maternal Burnout

While our findings show that difficulty with childcare exhibits more variance than expected, our results are in line with the conclusions of other researchers who claim that parental burnout is connected to the child’s characteristics and, in the case of children with a disability or chronic disease, to the severity of the disorder [5,32] such as serious dysfunction in motor skills, poor or deteriorating health, communication problems, and associated behavioral disorders [33]. We emphasize two main indicators of childcare difficulty as subjectively indicated by the mothers’ responses: a low degree of independence and self-care exhibited by the child and a low expectation of future rehabilitation. However, one must take into consideration the subjectivity of the evaluations and the minimalist format of the description of the level of the child’s functioning; these may underlie their relatively poor correlation with parental burnout in the analysis and their seeming lack of correlation with the family’s functionality. Quite possibly, a more extended and objectified evaluation of the child’s level of functioning, e.g., one similar to the Gilliam Autism Rating Scale, would improve how our models explain this relationship [34], especially since a connection between a child’s state and parental stress was revealed in Bravo’s (2005) [32] study. Note, however, that in other studies, such as the meta-analysis carried out by Cousino and Hazen (2009) [35], it was rated as insignificant. These researchers determined that while parents of children with chronic diseases demonstrate a level of parental stress significantly higher than parents of healthy children, in the sub-group of parents with chronically ill children, there was no significant correlation between parental stress and the condition’s duration or severity. On the other hand, positive correlations between parental stress and parental involvement in treatment or rehabilitation and between stress and a low level of psychological adjustment (for both parents and children) have been demonstrated [35].

### 4.2. Personality and Parental Burnout/Influence—Neuroticism and Conscientiousness

The findings showed a significant correlation between maternal emotional instability (i.e., neurotic disposition) and high conscientiousness, both of which lead to communication problems and family imbalance, leading in turn to dissatisfaction with family life. Both models demonstrated that these maternal personality traits had a stronger impact on parental burnout and the extent of family function than childcare difficulties.

This latter observation moves the discussion toward the context of personality-related conditioning. The results of our research are partly aligned with other studies. For example, Vigouroux et al. (2017) and Vigouroux et al. (2018) discovered that three features of the Big Five are of key importance [12,36]. These are neuroticism (versus emotional stability), agreeableness (to a large extent), and conscientiousness (although this was shown to be somewhat ambiguous).

The connection between neurotic disposition and burnout seemed to be quite obvious: this trait is associated with negative emotionality and high reactivity, and according to Alarcon et al. (2009), negative emotions are detrimental to developing and maintaining a positive relationship with the child. In fact, the connection between neuroticism and burnout was already confirmed in a 2009 meta-analysis of professional burnout [37].

In research carried out by Vigouroux et al. (2018), agreeability was inversely correlated with parental burnout with respect to both sub-dimensions (friendliness and cooperation) of the trait [12]. According to those authors, agreeability refers to attributes beneficial to the child, such as an inclination toward increased identification with their needs. In our research, we found no confirmation of such a correlation; based on the path analyses, the predictive power of agreeableness against burnout proved insignificant, although simple correlation analyses showed that maternal agreeableness was positively correlated with the degree of parental burnout. Interestingly, agreeableness also correlated positively with indicators of an effective family system. On the other hand, the correlation between maternal agreeableness and indicators of quality family communication, a better balance of the family system, and satisfaction with family life were negative. For this reason, when performing the path analyses, we disregarded agreeableness because it seemed to be a poor predictor of parental burnout and effective family communication.

The third trait is conscientiousness. A meta-analysis by Alarcon et al. (2009) suggested that conscientiousness protects one from (professional) burnout [37]. However, Vigouroux et al. (2018) concluded that the correlation between conscientiousness and burnout is complex: the two sub-components of this trait correlate with parental burnout in opposite directions, and therefore the correlation of the general indicator is close to zero [12]. However, our research led to entirely different conclusions. A high level of conscientiousness proved a significant predictor of the extent of parental burnout; furthermore, it was negatively correlated with family function (i.e., quality of communication and a balanced family system) in both path models. In model 2, conscientiousness also correlated negatively to maternal satisfaction with family life. This suggests that in families with children with autism, conscientiousness does not promote maternal welfare or family functionality and may contribute to dysfunctions in either area. The reason for this might be that high degrees of conscientiousness may, for example, reflect excessive submissiveness, or that conscientiousness may not necessarily mean self-control and the ability to organize one’s activity but rather an inclination toward perfectionism or a sense of guilt, both of which are predispositions to parental burnout [8].

### 4.3. The Influence of Family Function

The results herein generally corroborate those of surveys that have revealed a relationship between parental burnout and the functionality of the family system [4,32,38]. In Olson’s (2011) model, effective communication in the family is a factor in the remaining dimensions of family functionality: A balanced family system and satisfaction with family life [19]. Both of our models corroborated this theory. Interestingly, they poorly explained how the quality of communication affects the family (correlation was only 13% and 17%, respectively). According to Mikolajczak et al. (2018), burnout is negatively correlated with both marital satisfaction and the organization of the family system [4]. The correlations between burnout and co-parenting subscales in their paper also pointed to the important role played by distorted family communication in parental burnout. The authors concluded that family dysfunction (expressed as lack of agreement on childcare, exposure to family conflicts, a lack of marital satisfaction, and signs of disorganized family life) is a discernable precursor to parental burnout. While its influence is independent of the impact of personality traits, it has a similar intensity.

In our research into families with a child with autism, parental burnout was similarly related to a low level of functionality. However, our path analysis demonstrated an equally good adjustment of the model in which the above-mentioned causality variable is assumed to be an adjustment of the alternative model, assuming the opposite direction of the influence. In other words, parental burnout influences the quality of family communication, organization, and satisfaction. Thus, one cannot rule out either of the causality variables as being valid; the effects of stress/parental burnout and family functionality are circular in nature. This interpretation concurs with a systemic approach to family quality. It provides an important perspective because it shows alternative ways of influencing therapy or family support. By effectively preventing stress and parental burnout, family functionality can be reinforced in various areas (e.g., communication, organization of family life, care, and rehabilitation) [39,40]. At the same time, by modifying the functioning of the family system, one can expect beneficial changes in the parents’ disposition and an increase in their ability to cope with problems.

### 4.4. Limitations of the Study

The present study has several limitations. The first is the use of self-report questionnaires, which are prone to over or underestimation of the effect of the variables studies. Second, the participants were restricted to Polish mothers of children with autism, and there was no comparison group. Therefore, future research should include a control group such as fathers, mothers of children with other intellectual disabilities, or parents (mothers/fathers) of children with autism from other cultures. Third, we purposely excluded from the study (and from the models) stressors that people face in other areas of their lives (e.g., work stress, conflicts with extended family or neighbors, beliefs, and other major life events). There are so many of these that it would have been impossible to consider them all. Fourth, the models did not consider the wider context in which the parents live (e.g., more or less advantaged communities and cultural values). Finally, the burnout inventory is new to Poland; it, therefore, needs to be used with different populations.

### 4.5. Recommendations for Further Research and Clinical Implications

The limitations of the present study leave ample room for future researchers to probe and refine their findings. In addition to conducting cross-cultural research to identify macro systemic antecedents of parental burnout, cross-lagged longitudinal analyses and experimental intervention research is warranted. This would refine our understanding of causality links and processes among all types of parents. Future studies might also concentrate on uncovering antecedents that play a specific role in specific categories of parents; Lindahl Norberg et al. (2014) already conducted this research among parents with chronically and/or severely ill children [15]. Lindström et al. (2015) suggested that parents whose child’s future is uncertain because of the disease have a high need for control, and this may be a vulnerability factor for burnout [5]. Burnout may have specific antecedents in addition to the more general factors examined herein among other categories of parents (single parents, step-parents, LGBT parents, and so on). Refining the antecedent model for each category may help clinicians focus on the appropriate factors in each case.

The findings of the present study have important implications for clinical approaches. First, coping strategies (e.g., a problem-solving/optimistic approach) are an important factor in reducing parental burnout. Thus, practitioners need to encourage mothers to use these. Second, social support can reduce burnout, so it is important that mothers are encouraged to become involved in social support groups and share their experiences. This may help them prepare for future challenges. Third, having a supportive co-parent is important, so showing couples how they can support each other is another strategy that could be implemented in clinical settings. Finally, community intervention programs could be developed to improve the parents’ social support networks, not only to support and educate them in relation to possible difficulties with their children but also to teach them about their rights and to inform them about the available social resources. By adopting the view that prevention is the best medicine, educating mothers in stress reduction techniques and teaching them how to solve problems should ultimately lead to reduced burnout.

## 5. Conclusions

Path analysis revealed similar fit indexes for both models: (a) burnout is a mediator between exogenous variables and family functioning, and (b) family functioning is an indirect mediator between exogenous variables and burnout. In both models, both personality traits proved to be antecedents of the presence (or not) of effective family communication and a balanced family system. Emotional stability was positively related to effective communication and a balanced family system, whereas high conscientiousness was negatively related. (i.e., maternal emotional stability supported the family’s balance while her conscientiousness limited it.) These findings suggest that increased maternal emotional instability (neuroticism) and conscientiousness can lead to increased family communication problems, which may further lead to a breakdown of the equilibrium in the family system, resulting in the mother’s dissatisfaction with family life and a consequent increased risk of maternal burnout.

## Figures and Tables

**Table 1:** Respondents’ characteristics.

Variable		Value
*n*		410
Respondents’ age, mean (*SD*)		39.03 (7.42)
Respondents’ education levels, *n* (%)		
	Primary school	8 (1.95)
	Vocational	29 (7.07)
	Secondary	131 (31.95)
	Higher	242 (59.03)
Residence, *n* (%)		
	Rural areas	86 (20.98)
	Town < 10,000 inhabitants	25 (6.10)
	Town < 50,000 inhabitants	70 (17.07)
	City < 100,000 inhabitants	61 (15.12)
	City > 100,000 inhabitants	168 (40.98)
Family type, *n* (%)		
	Both biological parents	316 (77.07)
	Single parenthood	61 (15.13)
	Reconstructed family	33 (8.05)
ASD child age, mean (*SD*)		9.74 (7.41)
ASD child gender, *n* (%)		
	Male	299 (72.93)
	Female	111 (27.07)
Children’s autism dynamics, *n* (%)		
	Expectation of rehabilitation amenability	299 (72.93)
	Rehabilitation not expected	74 (18.05)
	Deterioration despite rehabilitation	37 (9.02)
Degree of children’s autism, *n* (%)		
	Mild disability	53 (12.93)
	Significantly reduced self-care	201 (49.02)
	Incapable to function independently	156 (38.05)
Currently employed, *n* (%)		131 (31.95)
Guardian with disabilities, *n* (%)		33 (8.05)
Parental burnout, mean (*SD*)		28.03 (6.62)
Family equilibrity, mean (*SD*)		0.00 * (1.00)
Family communication, mean (*SD*)		36.23 (8.52)
Satisfaction with family, mean (*SD*)		35.32 (9.29)

ASD: Autism Spectrum Disorder; *SD*: standard deviation; * standardized measure (*Z-score*).

**Table 2:** Correlation Matrix and Scales Reliability.

Variable	Dif	Ext	Agr	Con	Sta	Int	Bur	Equ	Comm	Sat
Difficult childcare (Dif)	1	−0.06	0.01	−0.02	−0.14 *	−0.04	0.24 *	−0.10 *	−0.10 *	−0.09
Extroversion (Ext)		1	−0.32 *	−0.08	0.36 *	0.09	−0.32 *	0.20 *	0.21 *	0.29 *
Agreeableness (Agr)			1	0.15 *	−0.21 *	−0.05	0.20 *	−0.27 *	−0.17 *	−0.22 *
Conscientiousness (Con)				1	−0.22 *	0.01	0.26 *	−0.25 *	−0.24 *	−0.29 *
Emotional stability (Sta)					1	−0.02	−0.68 *	0.42 *	0.31 *	0.40 *
Intellect/Imagination (Int)						1	0.01	0.14 *	0.03	0.05
Parental burnout (Bur)							1	−0.43 *	−0.36 *	−0.46 *
Family equilibrium (Equ)								1	0.61 *	0.64 *
Family communication (Com)									1	0.83 *
Satisfaction with family life (Sat)										1
Cronbach’s alpha	X	0.83	0.60	0.78	0.76	0.64	0.70	X	0.90	0.93

Note: * *p* < 0.05. X: coefficient was not calculated.

## Data Availability

Data are available from the corresponding author on a reasonable request.

## Supplementary Materials

The following are available online at https://www.mdpi.com/article/10.3390/ijerph19031187/s1: Table S1: Descriptive Statistics of Variables in the Data Set; Table S2: Test of the First Hypothetical Model with the Family Satisfaction as an Outcome Variable (All Possible Relationships in the “Saturated” Model); Table S3: Test of the Second Hypothetical Model with Parental Burnout as an Outcome Variable (All Possible Relationships in the “Saturated” Model); Table S4: Test of a Reduced First Hypothetical Model with Family Satisfaction as an Outcome Variable (After Removing Non-significant Relations from the Initial “Saturated” Model).
